# Comparison of complications and long-term survival after minimally invasive esophagectomy versus open esophagectomy in patients with esophageal cancer and chronic obstructive pulmonary disease

**DOI:** 10.3389/fonc.2022.934950

**Published:** 2022-10-04

**Authors:** Yu Rong, Yanbing Hao, Jun Xue, Xiaoyuan Li, Qian Li, Li Wang, Tian Li

**Affiliations:** ^1^ Department of Thoracic Surgery, The First Affiliated Hospital of Hebei North University, Zhangjiakou, China; ^2^ Department of General Surgery, The First Affiliated Hospital of Hebei North University, Zhangjiakou, China; ^3^ Department of Anesthesiology, The First Affiliated Hospital of Hebei North University, Zhangjiakou, China; ^4^ School of Basic Medicine, Fourth Military Medical University, Xi’an, China

**Keywords:** long-term survival, minimally invasive esophagectomy, open esophagectomy, esophageal cancer, chronic obstructive pulmonary disease (COPD)

## Abstract

**Objective:**

To compare the complications and long-term survival of esophageal cancer patients with chronic obstructive pulmonary disease (COPD) after minimally invasive esophagectomy (MIE) versus open esophagectomy (OE) using propensity score matching (PSM).

**Methods:**

Esophageal cancer patients who underwent esophagectomy at the Thoracic Surgery Department of the First Affiliated Hospital of Hebei North University from January 2010 to December 2018 were retrospectively enrolled. The incidence of postoperative complications and prognosis of the MIE (n = 132) and OE (n = 138) groups were compared. To reduce bias, 1:1 PSM was adopted for the analysis.

**Results:**

The median disease-free survival (DFS) of the MIE and OE groups were 24 months and 26 months, respectively, and neither group reached median survival. There was no significant difference between the two groups in terms of 3-year DFS and overall survival (OS). The stratification of the patients on the basis of the percentage of estimated forced expiratory volume in the first second (%FEV1) did not result in significant differences in the survival rates. A total of 42 patients (50%) in the MIE group and 55 patients (65.48%) in the OE group experienced complications, and the difference was statistically significant (OR=0.527, 95% CI: 0.283–0.981, P=0.042). The incidence of acute COPD exacerbation (OR=0.213, 95% OR, CI: 0.068–0.666, P=0.004) and pulmonary atelectasis requiring bronchoscopic aspiration (OR=0.232, 95% OR, CI: 0.082–0.659, P=0.004) were significantly higher in the OE versus the MIE group. In addition, the distribution of the various grades of complications also differed significantly between the two groups (P=0.016). While the incidence of minor complications (≤Grade II) was similar in both groups (P=0.503), that of severe complications (≥Grade III) was markedly higher in the OE group (P=0.002) and the Grade-IIIa complications were predominant (P=0.001). The severity of complications was correlated with the postoperative duration of hospital stay in both groups (r=0.187, P=0.015). No significant difference was observed in the incidence of minor complications (≤Grade II) between the two groups following stratification on the basis of %FEV1, whereas severe complications were more frequent in the OE group among patients with %FEV1 between 60% and 70% (P=0.001<0.05).

**Conclusion:**

There was no significant difference in the postoperative DFS and OS of esophageal cancer patients with COPD after undergoing MIE or OE. However, MIE significantly reduced the incidence of severe postoperative complications among patients with %FEV1 between 60% and 70%.

## Introduction

Cancer remains the main killer worldwide (1–4). Esophageal cancer is the sixth most commonly diagnosed cancer worldwide and accounts for 450,000 deaths annually (5–7). China is among the countries with a high prevalence of esophageal cancer (8). At present, it is primarily treated by minimally invasive esophagectomy (MIE) and open esophagectomy (OE). Currently, the minimally invasive esophagectomy that is widely used in clinic includes Ivor–Lewis esophagectomy and McKeown esophagectomy (9). In addition, there are some minimally invasive surgical methods for esophageal cancer, such as mediastinoscopic esophagectomy (10). However, this surgical method is different from the traditional surgical approach. It needs to complete the dissection of the esophagus and lymph nodes in the very narrow mediastinum. It requires skilled surgical techniques for thoracic surgeons. Therefore, the number of hospitals that can skillfully perform this surgery is small. Therefore, the MIE mentioned in this study is only two surgical methods, Ivor–Lewis esophagectomy and McKeown esophagectomy, which are widely used in clinic. Studies show that MIE has similar or even better long-term outcomes compared to OE (11–13), and its medium-term outcomes include less tumor invasion and a higher quality of life (14). Moreover, there is evidence that MIE reduces the incidence of postoperative pulmonary complications (15, 16). On the other hand, some studies have reported a similar incidence of pulmonary complications after MIE and OE, although anastomotic fistula and reinterventions are more frequent after MIE (17, 18). The complications associated with esophagectomy have a significant impact on postoperative mortality (19).

Chronic obstructive pulmonary disease (COPD) is an independent risk factor of postesophagectomy pulmonary complications (20). According to the Global Initiative for Chronic Obstructive Lung Disease (GOLD) guidelines (21), COPD is defined as forced expiratory volume in the first second (FEV1)/forced vital capacity (FVC) <70% following the inhalation of bronchodilator agents. Due to the rapidly aging population, the number of patients with esophageal cancer presenting COPD is expected to increase (22). The multifactorial pathophysiology of COPD poses an additional challenge for thoracic surgeons to select the optimal surgical approaches for esophageal cancer patients with this comorbidity. Furthermore, the injection of artificial CO_2_ during laparoscopy may lead to CO_2_ retention and even hypercapnia. Owing to obstructed airflow during expiration, COPD patients experience increased difficulty in expelling CO_2_ compared to patients with normal lung function. Some surgeons prefer to avoid performing laparoscopy in patients with poor lung function in order to reduce the impact of pneumoperitoneum on respiration. In addition, some randomized controlled trials (RCTs) have excluded patients with poor lung function to obtain satisfactory experimental results (23). In routine clinical practice, however, surgeons must perform esophagectomies for patients with various comorbidities. Therefore, it is debatable whether the results of these RCTs, which reflect the outcomes of experienced surgeons operating on carefully selected patients in high-volume institutions, can be extrapolated to the real-world scenario.

Therefore, we conducted such a retrospective study, focusing on the population of esophageal cancer patients with COPD, which has not been focused on in previous studies (24, 25). At the same time, we also tried to explore for the incidence of complications and long-term survival of the two surgical methods under different pulmonary function classification levels in this population for the first time, which may provide a certain reference for the precise selection of surgical methods.

## Participants and methods

### Study participants

This study was approved by the Ethics Committee of the First Affiliated Hospital of Hebei North University (K2018075). The requirement for informed written consent was waived on account of the retrospective nature of the study. Esophageal cancer patients who underwent esophagectomy at our hospital from January 2010 to December 2018 were reviewed. The inclusion criteria were as follows: 1) a definite absence of metastases from the brain, liver, and other organs as per preoperative MRI and CT, 2) esophageal cancer with postoperative pathological stages IA–IVA, 3) presence of squamous cell carcinoma suggested by postoperative pathological biopsy, and 4) tested for preoperative pulmonary function. Patients with 1) a history of other malignancies in the previous five years, 2) FEV1/FVC >70% following the administration of bronchodilator agents, 3) stage T4 M1 tumors treated by emergency esophagectomy or stage-2 esophagectomy in case exploratory laparoscopy revealed the invasion of surrounding tissues that were unresectable by R0 or the presence of residual tumor cells confirmed by postoperative biopsies were excluded.

Esophageal cancer was staged in accordance with the eighth edition of esophageal cancer staging (26). A total of 577 patients underwent esophagectomy during this period, and the choice of MIE or OE was based on the preference of the patient or surgeon. A total of 82 patients did not undergo preoperative pulmonary function testing, 35 had been diagnosed with other malignancies in the previous 5 years, and 164 patients showed FEV1/FVC >70% following the administration of bronchodilator agents. In addition, exploratory laparoscopy suggested the invasion of surrounding tissues that were unresectable by R0 in 26 patients, and residual cancer was confirmed in these patients by postoperative pathological biopsy. After excluding the above cases, 270 patients were enrolled, including 138 in the OE group and 132 in the MIE group.

### Methods

The demographical information, preoperative examination results, surgical approaches, and postoperative pathological data of all patients were collected from the medical record database of the hospital.

Both patient groups underwent esophagectomy by transthoracic or transcervical anastomosis. All patients were routinely admitted to the intensive care unit for stabilization and extubation after surgery, and those with stable signs were transferred back to the general wards on the first day postsurgery. Pain pumps were used after surgery for patient-controlled analgesia. Electrolyte-containing fluid (500 ml in a nutrition tube) was administered 48 h after surgery, and an enteral nutrition solution was administered starting 96 h postsurgery. The thoracic drainage tube was removed once the patient was able to eat the liquid diet, and it was ascertained that the discharged fluid was less than 200 ml and showed no significant change compared to that before drainage. Supplementary feeding through a jejunal nutrition tube was continued after discharge.

### Follow-up

Follow-up was performed by a direct telephone questionnaire or using outpatient data during the 4th week and the 3rd, 6th, and 12th months after surgery. Two annual outpatient reviews were conducted from the second year onwards.

### Study endpoints

The primary endpoint of this study was the occurrence of surgery-related complications, and the secondary endpoints were 3-year disease-free survival (DFS) and 3-year overall survival (OS). The postoperative complications were graded on the basis of treatment using the Clavien–Dindo classification (CDC) system (27) as follows: Grade I—no requirement for treatments such as drugs, surgery, endoscopy, and radioactive interventions (antiemetics, analgesics, diuretics, electrolytes, and physiotherapy are allowed); Grade II—Grade-I complications requiring medication treatment; Grade IIIa—requiring surgery, endoscopy, or radiotherapy without general anesthesia; Grade IIIb—requiring surgery, endoscopy, or radiotherapy with general anesthesia; Grade IVa—single organ failure; Grade IVb—multiple organ failure; and Grade V—death. Pulmonary and other postoperative complications were included, and the Grade-II and lower complications were grouped as “mild” and those of Grade III and above as “severe”. The highest-grade complication occurring in a patient was recorded as the overall grade for that patient (24). Pneumonia was defined as a suspected respiratory infection requiring antibiotics and meeting one or more of the following criteria: 1) new expectoration or change in the nature of existing sputum, 2) new pulmonary invasion suggested by sternum or computed tomography or worsening of pulmonary invasion compared to the condition suggested by the original sternum, and 3) fever and/or white blood cell (WBC) count >12 × 10^9^/L. Bronchial asthma was defined as newly detected expiratory wheeze following treatment with bronchodilator agents. The acute exacerbation of COPD was defined as the worsening of respiratory symptoms, increased sputum production, dyspnea, asthma attacks, etc (28). Systemic sepsis was defined as the presence of a definite infectious lesion with two or more of the following symptoms: body temperature <36°C or >38°C, heart rate >90 beats/min, respiratory rate >20 breaths/min, PaCO_2_ <32 mmHg, WBC count <4,000/mm³ or >12,000/mm³, and immature neutrophils >10% (29). Pleural effusion requiring drainage was defined as a moderate or greater volume of pleural effusion for which thoracic drainage was performed clinically. Pneumothorax requiring drainage was defined as a moderate or greater volume of pneumothorax for which closed thoracic drainage was performed clinically. Acute respiratory distress syndrome (ARDS) was defined as arterial partial pressure of oxygen (PO_2_)/fraction of inspiration O_2_ (FiO_2_) <200, positive end-expiratory pressure > 5 cm H_2_O for more than 24 h (30). DFS was defined as the time between surgery and tumor recurrence or death from any cause and OS as the time between surgery and death from any cause.

### Statistical analysis

Continuous variables were expressed as mean ± standard deviation. Propensity score matching (PSM) was performed using multivariate logistic regression models based on age, sex, height, weight, body mass index (BMI), age-corrected comorbidity index (aCCI index), the number of acute COPD episodes, smoking, alcohol consumption, CAT score, tumor, lymphaden, and metastasis (TNM) stage, left ventricular ejection fraction, preoperative PO_2_, preoperative PCO_2_, preoperative FVC, preoperative FEV1, the percentage of the estimated preoperative FEV1 (%FEV1), FEV1/FVC, and preoperative albumin concentration. A 1:1 match was used for patients undergoing OE or MIE, and the match tolerance was set to 0.02.

Student’s t-test was used to compare continuous variables that conformed to normal distribution between the two groups, and the chi-square test was used for categorical variables. The incidence of cumulative survival events was estimated using the Kaplan–Meier method, and the differences between the two groups were assessed by the log-rank test. The hazard ratio for each event in both groups was assessed by a Cox proportional hazard model. Following PSM, continuous and categorical variables were compared using the paired t-test and McNemar test, respectively. Differences in cumulative event rates after PSM were analyzed using stratified Cox. P < 0.05 was considered statistically significant. To adjust the effect of complications on survival, two models were used, with model 1 being unadjusted and model 2 adjusting model 1 for a different complication grade. Similarly, HRs and the 95% CIs of DFS and OS in response to different surgical groups across different complication grade were estimated. All statistical analyses were performed using SPSS V version 26.0 (IBM, Armonk, NY, USA).

## Results

### Propensity score matching

A total of 132 patients underwent MIE, and 138 were treated by OE. COPD was suggested in all cases as per the preoperative pulmonary function tests. The patients in the MIE group were shorter and weighed less and had a history of more acute COPD exacerbations, higher CAT scores, worse preoperative pulmonary function, lower PO_2_, and higher PCO_2_. In addition, the proportion of TNM stages was different in the two groups (**Table 1**).

**Table 1:** Baseline data of both groups before propensity score matching (PSM).

Variable	MIE group (n = 132)	OE group (n = 138)	P-value
Age	62.89 ± 8.49	62.75 ± 8.78	0.9
Number of males (%)	117 (88.63)	125 (90.58)	0.601
Height	164.07 ± 7.07	166.94 ± 6.61	0.001
Weight	55.93 ± 6.30	58.730 ± 7.67	0.001
BMI	20.78 ± 1.98	21.06 ± 2.37	0.284
Number of AECOPD	1.33 ± 1.98	0.83 ± 1.49	0.022
Smoking (%)			0.197
Never	10 (7.58)	4 (2.90)	
Previously	21 (15.91)	20 (14.49)	
Still smoking	101 (76.52)	114 (82.61)	
Drinking alcohol (%)			0.727
Never	30 (22.73)	28 (20.29)	
Previously	27 (20.45)	25 (18.12)	
Still drinking alcohol	75 (56.82)	85 (61.59)	
Left ventricular ejection fraction	61.64 ± 3.86	61.85 ± 4.49	0.679
Preoperative pulmonary function			
Preoperative FVC	2.74 ± 0.43	2.89 ± 0.51	0.018
Preoperative FEV1	1.70 ± 0.30	1.84 ± 0.36	0.001
Preoperative FV1/FVC	61.94 ± 6.40	63.84 ± 5.33	0.009
Preoperative FEV1 as a percentage of the estimate	64.96 ± 7.63	66.83 ± 7.55	0.044
Preoperative PO_2_, mmHg	78.77 ± 10.22	81.28 ± 9.98	0.042
Preoperative PCO_2_, mmHg	38.77 ± 3.89	37.09 ± 1.91	<0.001
Preoperative CAT score	12.57 ± 3.34	11.71 ± 3.28	0.034
Preoperative albumin concentration, g/L	41.47 ± 3.44	41.12 ± 3.44	0.399
Number of cigarettes used per year	840.91 ± 504.11	727.17 ± 463.88	0.055
aCCI index	1.44 ± 0.69	1.51 ± 0.78	0.449
TNM stage (%)			0.018
I	46 (34.85)	37 (26.81)	
II	43 (32.58)	30 (21.74)	
III	36 (27.27)	58 (42.03)	
IV	7 (5.30)	13 (9.42)	

**Figure 1:**
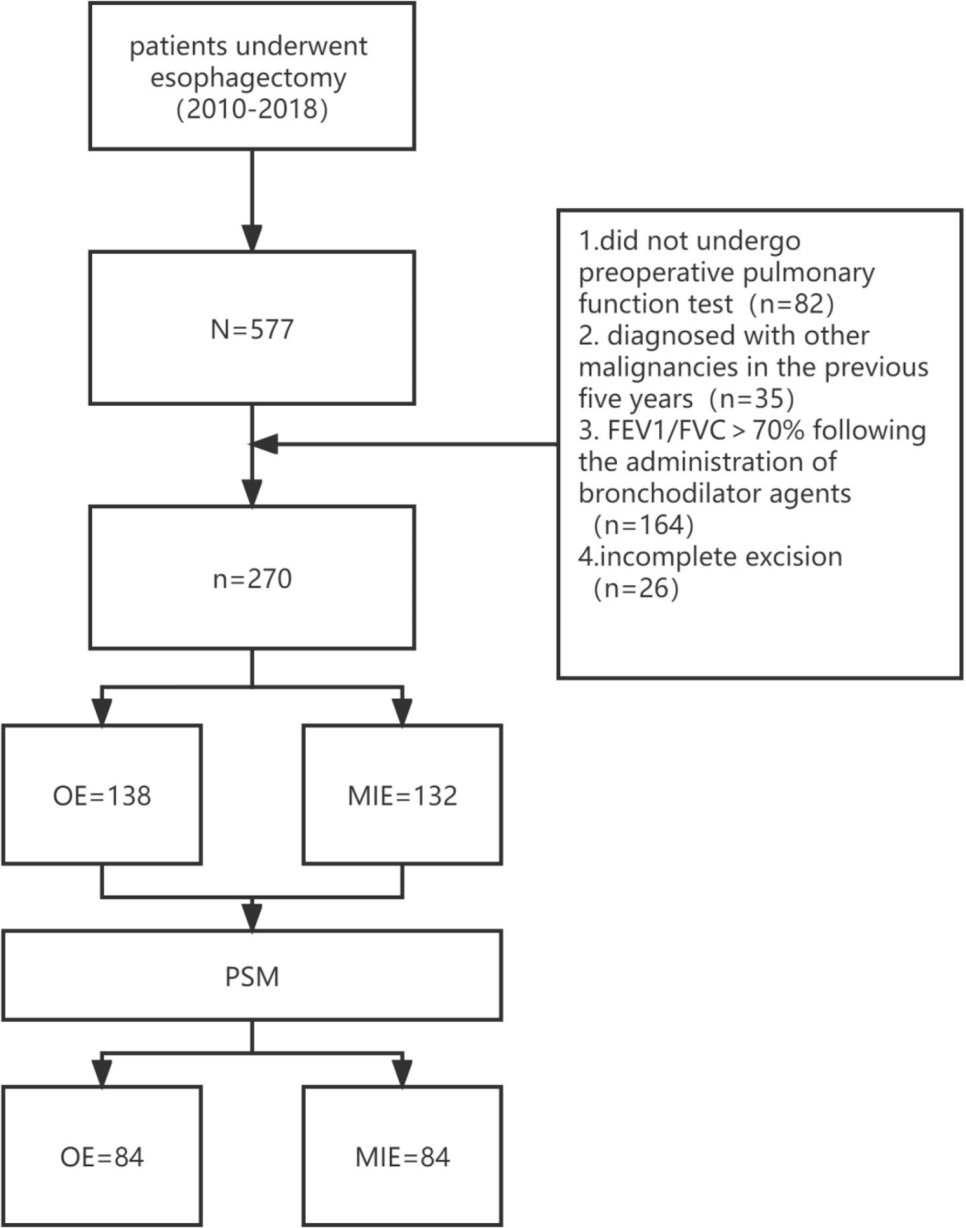
Flow diagram for patient selection.

In total, 84 pairs of patients were successfully matched after PSM. A flow diagram is shown in **Figure 1**. No significant difference was observed between the two groups in terms of height, weight, a history of hypertension, the number of acute COPD episodes, CAT scores, preoperative pulmonary function, PO_2_, and PCO_2_. In addition, the proportion of the TNM stages was similar (**Table 2**).

**Table 2:** Baseline data after PSM.

Variable	MIE group (n=84)	OE group (n=84)	P-value
Age	64.15 ± 7.33	62.79 ± 8.99	0.281
Number of males (%)	74 (88.10)	76 (90.48)	0.804
Height	165.71 ± 7.48	165.19 ± 5.89	0.616
Weight	57.08 ± 6.58	56.95 ± 6.48	0.894
BMI	20.79 ± 2.01	20.89 ± 2.33	0.76
Number of AECOPD	0.99 ± 1.41	0.70 ± 1.45	0.197
Smoking (%)			0.673
Never	4 (4.76)	3 (3.57)	
Previously	12 (14.29)	17 (20.24)	
Still smoking	68 (80.95)	64 (76.19)	
Drinking alcohol (%)			0.602
Never	20 (23.81)	18 (21.43)	
Previously	17 (20.24)	13 (15.48)	
Still drinking alcohol	47 (55.95)	53 (63.10)	
Left ventricular ejection fraction	61.56 ± 3.84	61.55 ± 4.37	0.985
Preoperative pulmonary function			
Preoperative FVC	2.78 ± 0.43	2.84 ± 0.50	0.414
Preoperative FEV1	1.75 ± 0.31	1.81 ± 0.35	0.257
Preoperative FV1/FVC	63.15 ± 5.71	63.93 ± 5.30	0.364
Preoperative FEV1 as a percentage of the estimate	66.28 ± 6.27	67.94 ± 7.81	0.131
Preoperative PO_2_, mmHg	80.80 ± 10.59	80.52 ± 10.45	0.866
Preoperative PCO_2_, mmHg	37.44 ± 2.89	37.37 ± 1.98	0.852
Preoperative CAT score	11.96 ± 2.72	11.08 ± 3.10	0.052
Preoperative albumin concentration, g/L	41.89 ± 3.39	41.74 ± 3.36	0.767
Number of cigarettes used per year	772.38 ± 376.27	710.71 ± 438.80	0.33
aCCI index	1.45 ± 0.75	1.44 ± 0.81	
TNM stage (%)			0.149
I	27 (32.14)	28 (33.33)	
II	27 (32.14)	21 (25.00)	
III	23 (27.38)	33 (39.29)	
IV	7 (8.33)	2 (2.38)	

### Postoperative complications in both groups

A total of 42 patients (50%) in the MIE group and 55 (65.48%) in the OE group experienced complications, and the difference between the two groups was statistically significant (OR=0.527, 95% CI: 0.283–0.981, P=0.042). The most frequent complication in the OE group was pneumonia (26.19%) followed by pulmonary atelectasis requiring bronchoscopy (21.43%), acute exacerbation of COPD (19.05%), pleural effusion requiring additional drainage (16.67%), thoracic incision dehiscence (15.48%), recurrent laryngeal nerve injury (8.33%), arrhythmia requiring intervention (8.33%), anastomotic fistula (7.14%), bronchial asthma (7.14%), pneumothorax requiring reintubation (7.14%), ARDS (5.95%), congestive heart failure requiring intervention (2.38%), gastroparesis (2.38%), pyloric obstruction (1.19%), chylothorax (1.19%) and systemic sepsis (1.19%) in that order. Pneumonia was also the most common complication (19.05%) in the MIE group followed by anastomotic fistula (14.29%), recurrent laryngeal nerve injury (9.52%), thoracic incision dehiscence (8.33%), pleural effusion requiring additional intubation (7.14%), pulmonary atelectasis requiring bronchoscopy (5.95%), arrhythmia requiring intervention (5.95%), acute exacerbation of COPD (4.76%), bronchial asthma (3.57%), gastroparesis (1.19%), pyloric obstruction (1.19%), congestive heart failure requiring intervention (1.19%), chylothorax (1.19%), and pneumothorax requiring reintubation (1.19%) (**Table 3**).

**Table 3:** Postoperative complications in the minimally invasive esophagectomy (MIE) and open esophagectomy (OE) groups.

Postoperative complications	MIE group, n (%)	OE group, n (%)	OR	95% CI (low limit)	95% CI (high limit)	P-value
Recurrent laryngeal nerve injury	8 (9.52)	7 (8.33)	1.158	0.400	3.351	0.787
Pneumonia	16 (19.05)	22 (26.19)	0.663	0.319	1.376	0.269
Anastomotic fistula	12 (14.29)	6 (7.14)	2.167	0.773	6.075	0.134
Gastroparesis	1 (1.19)	2 (2.38)	0.494	0.044	5.554	1.000
Pyloric obstruction	1 (1.19)	1 (1.19)	1.000	0.062	16.256	1.000
Arrhythmia requiring intervention	5 (5.95)	7 (8.33)	0.696	0.212	2.288	0.549
Congestive heart failure requiring intervention	1 (1.19)	2 (2.38)	0.494	0.044	5.554	1.000
Bronchial asthma	3 (3.57)	6 (7.14)	0.481	0.116	1.993	0.496
Acute exacerbation of COPD	4 (4.76)	16 (19.05)	0.213	0.068	0.666	0.004
Thoracic incision dehiscence	7 (8.33)	13 (15.48)	0.497	0.188	1.315	0.153
Chylothorax	1 (1.19)	1 (1.19)	1.000	0.062	16.256	1.000
Pulmonary atelectasis requiring bronchoscopy	5 (5.95)	18 (21.42)	0.232	0.082	0.659	0.004
Pneumothorax requiring reintubation	1 (1.19)	6 (7.14)	0.157	0.018	1.331	0.117
Pleural effusion requiring additional drainage	6 (7.14)	14 (16.67)	0.385	0.140	1.055	0.057
ARDS	1 (1.19)	5 (5.95)	0.190	0.022	1.666	0.210
Systemic sepsis	1 (1.19)	1 (1.19)	1.000	0.062	16.256	1.000

**Figure 2:**
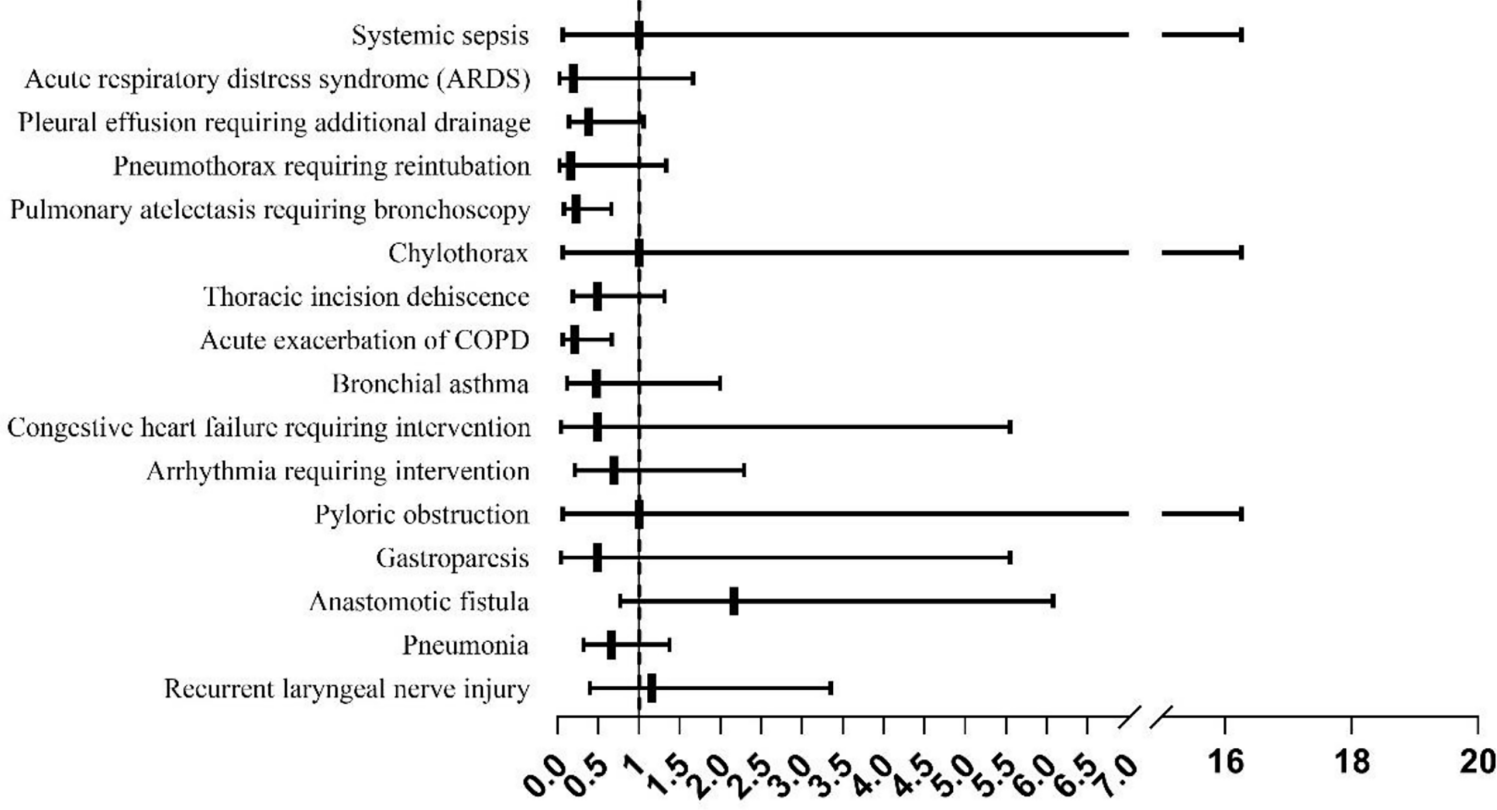
The incidence of complications in the minimally invasive esophagectomy (MIE) and open esophagectomy (OE) groups.

The frequency of acute COPD exacerbation (OR=0.213, 95% OR, CI: 0.068–0.666, P=0.004) and pulmonary atelectasis requiring bronchoscopic aspiration (OR=0.232, 95% OR, CI: 0.082–0.659, P=0.004) were significantly higher in the OE group compared to that in the MIE group (**Figure 2**).

**Figure 3:**
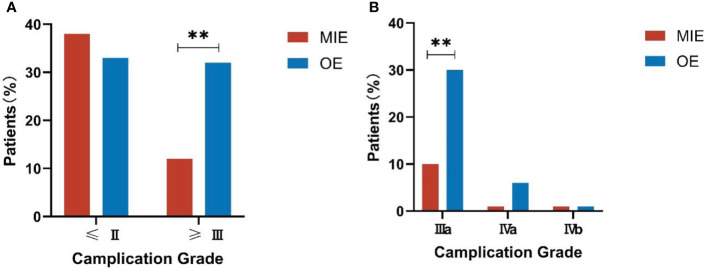
Comparison of the complication grades between both groups. **(A)** Incidence of serious complications (≥Grade III). **(B)** Incidence of Grade-IIIa complications. **P<0.01.

The complications were graded according to the CDC as described in the methods section. The distribution of complication grades between the two groups was significantly different (P=0.016, see **Table 4**). While the incidence of minor complications (≤Grade II) was similar in both groups (P=0.503), serious complications (≥Grade III) were significantly more frequent in the OE group compared to the MIE group (P=0.002), and the Grade-IIIa complications were predominant in the former (P=0.001). No deaths occurred in either group after matching (**Figure 3**). Furthermore, the mean postoperative hospital stay was significantly shorter for the MIE group compared to the OE group (12.52 ± 1.65 days vs. 13.35 ± 1.18 days; P=0.038). In both groups, the severity of complications was correlated with the duration of postoperative hospital stay (r=0.187, P=0.015).

**Table 4:** Distribution of complications classified into different grades.

Postoperative complications	MIE group, n (%)	OE group, n (%)	OR	95% CI (low limit)	95% CI (high limit)	P-value
Overall incidence of complications	42 (50.00)	55 (65.48)	0.527	0.283	0.981	0.042
Clavien–Dindo classification						0.016
≤Grade II	32 (38.10)	28 (33.33)	0.789	0.394	1.579	0.503
≥Grade III	10 (11.90)	27 (32.14)	0.256	0.108	0.608	0.002
Grade IIIa	8 (9.523810%)	25 (29.76)	0.248	0.105	0.590	0.001
Grade IIIb	0	0	NA	NA	NA	NA
Grade IVa	1 (1.19)	5 (5.95)	0.190	0.022	1.666	0.210
Grade IVb	1 (1.19%)	1 (1.19)	1.000	0.062	16.256	1.000
Grade V	0	0	NA	NA	NA	NA

NA, not applicable.

Based on the %FEV1 after matching, both groups were further divided into the ≤50%, 50%–60%, 60%–70%, 70%–80%, and >80% subgroups. In the MIE group, 11 (13.09%), 54 (64.29%), 16 (19.05%), and 3 (3.57%) patients had %FEV1 50%–60%, 60%–70%, 70%–80%, and >80%, respectively. In the OE group, 1 (1.19%), 7 (8.33%), 53 (63.10%), 16 (19.05%), and 7 (8.33%) patients were classified into the 50%–60%, 60%–70%, 70%–80% and >80% %FEV1 subgroups, respectively. The incidence of complications was significantly lower in the MIE patients with %FEV1 between 60% and 70% compared to the OE patients in the same subgroup (χ2 = 7.023, P=0.008 < 0.05) (**Table 4**).

### Impact of minimally invasive esophagectomy and open esophagectomy on patient survival

**Figure 4:**
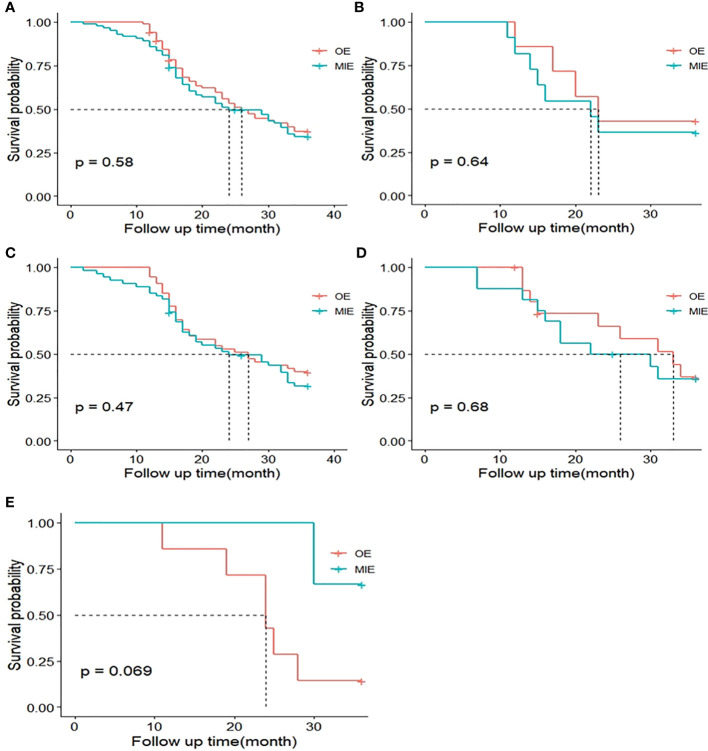
Kaplan–Meier curves of 3-year disease-free survival (DFS) between both groups. **(A)** DFS curves of the MIE and OE groups after matching. **(B)** DFS curves of patients with the percentage of estimated forced expiratory volume in the first second (%FEV1) 50%–60%. **(C)** DFS curves of patients with %FEV1 60%–70%. **(D)** DFS curves of patients with %FEV1 70%–80%. **(E)** DFS curves of patients with %FEV1 >80%.

The median postoperative DFS in the OE and MIE groups were 26 and 24 months, respectively, and there was no statistically significant difference between the two groups (p=0.580). To adjust the effect of complications on survival, two models were used, with model 1 being unadjusted and model 2 adjusting model 1 for a different complication grade. Similarly, the HRs and 95% CIs of DFS and OS in response to different surgical groups across different complication grades were estimated. When a different complication grade was used as a covariate in the fully adjusted model (model 2), MIE and OE did not appear to have an benefit in terms of improving DFS (HR: 1.03; 95%CI: 0.61–1.77; P=0.90) and OS (HR: 1.06; 95% CI: 0.71–1.57; P=0.78, **Table 5**). In patients without complications, MIE and OE did not appear to have an benefit in terms of improving DFS (HR: 0.97; 95% CI: 0.46-2.04; P=0.94) and OS (HR: 1.02; 95% CI: 0.57-1.85; P=0.94). In patients with minor complications, MIE and OE did not appear to have an benefit in terms of improving DFS (HR: 1.21;95% CI: 0.51–2.87;P=0.67) and OS (HR: 1.12;95% CI: 0.58–2.17; P=0.74). In patients with serious complications, MIE and OE did not appear to have an benefit in terms of improving DFS (HR: 0.69;95% CI: 0.09-5.40; P=0.73 and OS (HR: 1.02;95% CI: 0.57–1.85; P=0.94, **Table 6**). The patients were further stratified on the basis of %FEV1, and only one patient in the OE group could not be statistically analyzed in the ≤50% subgroup. As shown in **Figure 4**, the DFS rates of the MIE and OE groups did not show any significant difference in the 50%–60% (P=0.643), 60%–70% (P=0.469), 70%–80% (P=0.685) and >80% (P=0.069) %FEV1 subgroups.

**Table 5:** Different surgical groups and the risk of outcomes.

Outcome	Model 1		Model 2	
	HR (95%CI)	P-value	HR (95%CI)	P-value
DFS	1.00 (0.61, 1.65)	0.99	1.03 (0.61, 1.77)	0.90
OS	1.03 (0.70, 1.50)	0.89	1.06 (0.71, 1.57)	0.78

Model 1 was unadjusted.

Model 2 was adjusted for different complication grades.

**Table 6:** Effect of MIE on disease-free survival and overall survival in participants with different complications grade relative to OE.

	N	DFS			OS		
Complications		HR	95%CI	P-value	HR	95%CI	P-value
None	71	0.97	0.46-2.04	0.94	1.02	0.57-1.85	0.94
Minor (≤Grade II)	60	1.21	0.51-2.87	0.67	1.12	0.58-2.17	0.74
Serious (≥Grade III)	37	0.69	0.09-5.40	0.73	1.02	0.57-1.85	0.94

Outcome, DFS/OS; Exposure, OE/MIE.

**Figure 5:**
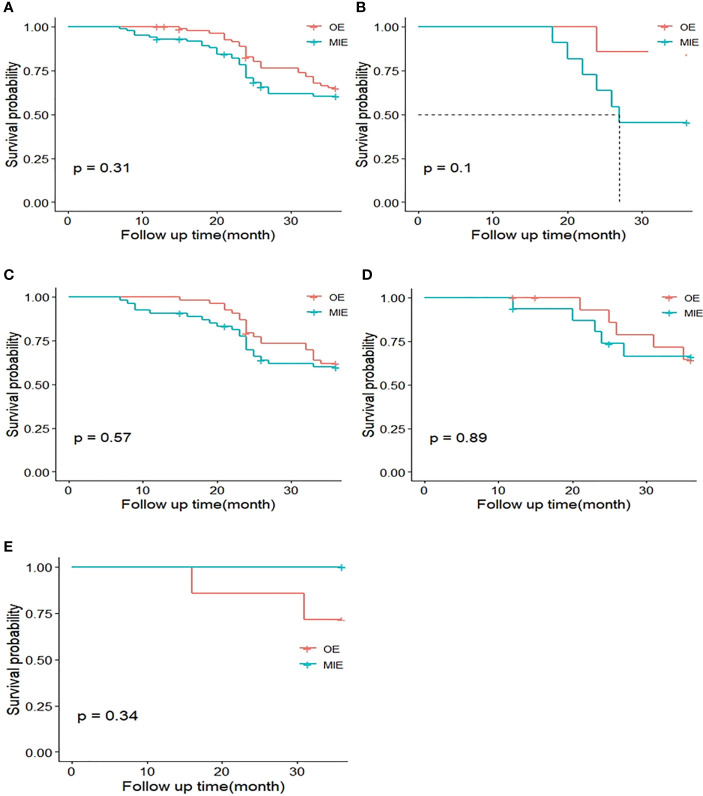
Kaplan–Meier curves of 3-year overall survival (OS) between both groups. **(A)** OS curves of the MIE and OE groups after matching. **(B)** OS curves of patients with %FEV1 50%–60%. **(C)** OS curves of patients with %FEV1 60%–70%. **(D)** OS curves of patients with %FEV1 70%–80%. **(E)** OS curves of patients with %FEV1 >80%.

Neither the MIE nor the OE group achieved median survival (P=0.341), and the OS was similar in both groups. As with DFS analysis, the patients were stratified by %FEV1 and only one patient in the OE group was not included in the ≤50% subgroup. The P-values for the OS rates in the 50%–60%, 60%–70%, 70–80%, and >80%FEV1 subgroups were 0.101, 0.575, 0.886, and 0.335, respectively (**Figure 5**), indicating the lack of any significant difference.

## Discussion

There were three major findings for this study: 1) MIE did not improve the 3-year DFS or OS compared to OE in esophageal cancer patients with COPD, even after stratification on the basis of pulmonary function (%FEV1); 2) the overall incidence of complications and that of severe complications were lower in the MIE group compared to the OE group, whereas the minor complications were similar in both groups; and 3) following stratification on the basis of the severity of COPD, patients in the OE group with %FEV1 60%–70% were at a higher risk of complications. In conclusion, MIE can reduce the risk of surgical complications and achieve the same long-term survival as OE in esophageal cancer patients with COPD.

Several studies have evaluated the therapeutic efficacy of MIE and OE against esophageal cancer (12, 13, 15, 31, 32) and compared the short-term and/or long-term outcomes of MIE and conventional surgery. While both MIE and OE can effectively prolong long-term patient survival (11, 25, 33), one study reported better outcomes following MIE (13). Consistent with previous findings, postoperative DFS and OS were similar in the MIE and OE groups of our cohort, which is indicative of the long-term efficacy of MIE. Even after stratifying the patients in each group on the basis of lung function, MIE or OE had no significant impact on postoperative DFS and OS. The first RCT comparing the short-term outcomes of MIE and OE reported that MIE reduced the incidence of pulmonary infections, shortened the hospital stay, and improved the quality of life 2 weeks after surgery and during hospitalization, without affecting the quality of the resected specimen (34). A recent systematic review and meta-analysis further showed that MIE has more favorable short-term outcomes compared to OE (35) and is therefore increasingly being considered for treating esophageal cancer. These studies, however, failed to focus on patients with COPD as a comorbidity.

COPD or decreased lung function is an independent risk factor of pulmonary complications following esophagectomy (20). Almost 50% of esophageal cancer patients presenting COPD develop pneumonia after surgery (36). Although the incidence of pneumonia was not particularly high in our cohort, it was still the most frequent complication in both surgical groups. A 2006 study on the postoperative pulmonary complications in esophageal cancer patients with COPD did not compare the outcomes of conventional surgery and MIE since the latter was not performed frequently at that time (37). In addition, thoracic surgeons often hesitate to perform MIE since it requires artificial pneumothorax and pneumoperitoneum that use CO_2_. An early study found that this non-physiological alteration of pneumoperitoneum resulted in the cephalad displacement of the diaphragm due to increased intra-abdominal pressure and consequent compressional dysplasia of the lower lobes of both lungs (38). In addition, there is evidence that pneumoperitoneum might lead to CO_2_ retention and subsequent hypercapnia (39). In patients without COPD, these effects can be modulated by adjusting the intraoperative breathing pattern through the administration of anesthesia (40), which is associated with few significant adverse consequences. In COPD patients, however, failure to expel intraoperative CO_2_ due to the reduced gas exchange function of lungs can lead to hypercapnia, resulting in multiple postoperative complications that compromise postoperative recovery (41).

Given the complex pathophysiology of COPD, it is challenging to determine the optimal surgical approaches for patients with pulmonary disease. Most surgeons and anesthesiologists prefer open surgery for COPD patients. Despite the potential negative physiological changes resulting from pneumoperitoneum, clinical outcomes increasingly show a lower incidence of pulmonary complications after laparoscopy. A propensity score analysis based on large databases compared the impact of COPD on the outcomes of laparoscopic and open surgery in six different surgical subgroups. Laparoscopic surgery reduced the incidence of respiratory, circulatory, and other multisystem complications compared to conventional open surgery (42). Another study showed that minimally invasive laparoscopic surgery reduced the risk of postoperative pulmonary and other complications, shortened the duration of hospital stay, and lowered the incidence of postoperative infection and deep venous thrombosis that can be fatal in COPD patients with poor physical condition. Thus, minimally invasive techniques should be recommended as the optimal surgical option for COPD patients (43). Consistent with the findings of Markar et al. (16) regarding the frequency of overall complications after MIE and OE, our study showed that MIE reduced the incidence of severe postoperative complications. However, the same group reported that MIE increased the prevalence of postoperative complications above Grade III, which contradicts our findings. In our study, the difference in the incidence of serious complications (≥ Grade III) is mainly due to the significant difference in the incidence of Grade IIIa complications. Although the complications at this level need to be treated without general anesthesia, after active and reasonable treatment, the patients will not continue to get worse or even die, which is quite different from that of Fransen et al. (24). This can be seen in the incidence of Grade-IVa, -IVb, and -V complications in this study. There is no significant difference between them. Therefore, although the incidence of serious complications in the OE group was higher than that in the MIE group, it did not affect the final prognosis of patients. Our adjusted model 2 strongly suggests that the complication grade does not affect the risk of prognoses.

We also found that the average duration of postoperative hospital stay was shorter in the MIE group compared to the OE group, and the severity of complications was positively correlated with the length of hospital stay. There were no cases of deep venous thrombosis in our cohort, most likely due to the routine postoperative administration of anticoagulants. We have also shown for the first time that MIE reduced the incidence of complications in COPD patients with %FEV1 between 60% and 70%, whereas no significant impact was seen among patients with worse or better lung function. Thus, MIE can be recommended as the optimal treatment option for COPD patients with %FEV1 60%–70%.

This study has several limitations that ought to be considered. First, the patient cohort was enrolled from a single center and analyzed retrospectively. Although PSM was used to avoid confounding factors between groups, some samples were lost after matching due to the small sample size, leading to possible bias in the results. Second, patient enrollment was challenging since only a small percentage fulfilled the inclusion criteria. Thus, the patients were only followed-up for 3 years, leading to the absence of 5-year DFS and OS data and resulting in less representative survival outcomes. In addition, there are many influencing factors for postoperative complications, such as postoperative pain that is known to cause patients to refuse to cough actively. That may increase the incidence of pulmonary complications (44). This retrospective study has been unable to obtain each patient’s postoperative pain score, resulting in no further adjustment of the confounders. We are ready to use a prospective cohort study in the future, including more patients and accurately recording perioperative indicators (including the nutritional status score and pain score). Standardized long-term follow-up will also be conducted to evaluate the advantages and disadvantages of MIE in the surgical treatment of esophageal cancer.

MIE and OE are equally effective in terms of long-term recurrence and survival in patients with esophageal cancer combined with COPD. However, MIE can reduce the incidence of severe postoperative complications, especially in COPD patients with %FEV1 between 60% and 70%.

## Data availability statement

The datasets presented in this study can be found in online repositories. The names of the repository/repositories and accession number(s) can be found below: https://www.jianguoyun.com/p/Dbhf2KwQgq2dChjyptoEIAA.

## Ethics statement

This study was approved by the Ethics Committee of the First Affiliated Hospital of Hebei North University (K2018075). The requirement for informed written consent was waived on account of the retrospective nature of the study.

## Author contributions

YR and YH conceived the study. YR and JX drafted the manuscript. XL and QL conducted statistical analysis on the data. YBH and TL revised the manuscript rigorously. LW collected the clinical data. All authors contributed to the article and approved the submitted version.

## Funding

This work was supported by the Youth Science and Technology Project of Scientific Research of Hebei Province (20180864).

## Conflict of interest

The authors declare that the research was conducted in the absence of any commercial or financial relationships that could be construed as a potential conflict of interest.

## Publisher’s note

All claims expressed in this article are solely those of the authors and do not necessarily represent those of their affiliated organizations, or those of the publisher, the editors and the reviewers. Any product that may be evaluated in this article, or claim that may be made by its manufacturer, is not guaranteed or endorsed by the publisher.

## Abbreviations

aCCI, age-corrected comorbidity index; ARDS, acute respiratory distress syndrome; BMI, body mass index; CAT, COPD Assessment Test; COPD, chronic obstructive pulmonary disease; CO2, carbon dioxide; CDC, Clavien–Dindo classification; CT, computed tomography; DFS, disease-free survival; FEV1, forced expiratory volume in the first second; %FEV1, percentage of estimated forced expiratory volume in the first second; FiO2, fraction of inspired oxygen; FVC, forced vital capacity; ICU, intensive care unit; MIE, minimally invasive esophagectomy; MRI, magnetic resonance imaging; OE, open esophagectomy; OS, overall survival; PCO_2_, partial pressure of carbon dioxide; PO_2_, Partial pressure of oxygen; PSM, propensity score matching; RCTs, randomized controlled trials; WBC, white blood cell.
